# Hyperacetylation of Cardiac Mitochondrial Proteins Is Associated with Metabolic Impairment and Sirtuin Downregulation after Chronic Total Body Irradiation of ApoE ^-/-^ Mice

**DOI:** 10.3390/ijms20205239

**Published:** 2019-10-22

**Authors:** Zarko Barjaktarovic, Juliane Merl-Pham, Ignacia Braga-Tanaka, Satoshi Tanaka, Stefanie M. Hauck, Anna Saran, Mariateresa Mancuso, Michael J. Atkinson, Soile Tapio, Omid Azimzadeh

**Affiliations:** 1 Institute of Radiation Biology, Helmholtz Zentrum München, German Research Center for Environmental Health GmbH, Institute of Radiation Biology, Ingolstädter Landstrasse 1, 85764 Neuherberg, Germany; Zarko.Barjaktarovic@helmholtz-muenchen.de (Z.B.); atkinson@helmholtz-muenchen.de (M.J.A.); soile.tapio@helmholtz-muenchen.de (S.T.); 2 Agency for Medicines and Medical Devices of Montenegro, 81000 Podgorica, Montenegro; 3 Research Unit Protein Science, Helmholtz Zentrum München, German Research Center for Environmental Health GmbH, 80939 München, Germany; juliane.merl@helmholtz-muenchen.de (J.M.-P.); hauck@helmholtz-muenchen.de (S.M.H.); 4 Institute for Environmental Sciences (IES), Rokkasho, Aomori 039-3213, Japan; tanakaib@ies.or.jp (I.B.-T.); tanakas@ies.or.jp (S.T.); 5 Laboratory of Biomedical Technologies, Agenzia Nazionale per le Nuove Tecnologie, l’Energia e lo Sviluppo Economico Sostenibile (ENEA), 76 00196 Rome, Italy; anna.saran@enea.it (A.S.); mariateresa.mancuso@enea.it (M.M.); 6 Chair of Radiation Biology, Technical University Munich, 80333 Munich, Germany

**Keywords:** ionising radiation, chronic exposure, TBI, acetylome, proteomics, sirtuins, heart, mitochondria, cardiovascular disease, PPAR alpha

## Abstract

Chronic exposure to low-dose ionizing radiation is associated with an increased risk of cardiovascular disease. Alteration in energy metabolism has been suggested to contribute to radiation-induced heart pathology, mitochondrial dysfunction being a hallmark of this disease. The goal of this study was to investigate the regulatory role of acetylation in heart mitochondria in the long-term response to chronic radiation. ApoE-deficient C57Bl/6J mice were exposed to low-dose-rate (20 mGy/day) gamma radiation for 300 days, resulting in a cumulative total body dose of 6.0 Gy. Heart mitochondria were isolated and analyzed using quantitative proteomics. Radiation-induced proteome and acetylome alterations were further validated using immunoblotting, enzyme activity assays, and ELISA. In total, 71 proteins showed peptides with a changed acetylation status following irradiation. The great majority (94%) of the hyperacetylated proteins were involved in the TCA cycle, fatty acid oxidation, oxidative stress response and sirtuin pathway. The elevated acetylation patterns coincided with reduced activity of mitochondrial sirtuins, increased the level of Acetyl-CoA, and were accompanied by inactivation of major cardiac metabolic regulators PGC-1 alpha and PPAR alpha. These observations suggest that the changes in mitochondrial acetylation after irradiation is associated with impairment of heart metabolism. We propose a novel mechanism involved in the development of late cardiac damage following chronic irradiation.

## 1. Introduction

Epidemiological studies show a clear causal association between increased risk of cardiovascular disease (CVD) and radiation exposure affecting tens of thousands of people annually [1,2]. Adverse effects of radiation exposure such as myocardial fibrosis and damage to the coronary arteries and microvasculature [3] were first observed at high doses in patients receiving radiotherapy for breast cancer, Hodgkin´s disease and a number of childhood cancers [4,5,6,7]. Recently, data have been accruing showing an increased risk of CVD even in occupationally exposed populations, such as nuclear workers, raising concerns about the risk of heart disease with doses accumulated at a low dose rate over many years [8,9,10,11,12]. The cumulative radiation doses in occupational settings vary considerably [13]. In Germany, for example, cumulative doses of up to 0.2 Gy for aircrew members are possible [13]. Among the Mayak nuclear workers, the mean cumulative external gamma dose was 0.51 Gy, with a maximum of 6.8 Gy [14]. Independent of the dose rate, doses of 0.5 Gy have been shown to result in a significant increase in CVD risk [2]. In contrast to high-dose-rate exposures, practically nothing is known about the biological mechanisms of CVD caused by chronic low-dose-rate exposures.

Our previous studies using C57Bl/6 mice and its ApoE-deficient (ApoE ^-/-^) derivative, exposed to local high-dose, high-dose-rate heart irradiation, suggested persistently decreased fatty acid oxidation (FAO) and inactivation of its transcriptional regulator, peroxisome proliferator-activated receptor (PPAR) alpha, in the irradiated heart [15,16]. This was associated with the reduced respiratory capacity of cardiac mitochondria [16,17]. Importantly, similar findings were observed in a study of cardiac autopsies from Mayak plutonium enrichment plant workers who were chronically exposed to external gamma rays at very low dose rates. Profiling of the cardiac proteome in the left ventricle of these workers showed dose-dependent inactivation of PPAR alpha and dysregulation of mitochondrial proteins at cumulative doses greater than 100 mGy [18].

Lysine acetylation of mitochondrial proteins has emerged as the key post-translational modification (PTM) in the metabolic control [19,20,21] and is one of the causal factors of metabolic derangements in various heart diseases [22,23]. In mitochondria, protein acetylation occurs via non-enzymatic and enzymatic pathways. In the non-enzymatic pathway, proteins are acetylated based on the availability of acetyl-coenzyme A (acetyl-CoA); this is the primary pathway of acetylation in mitochondria [22]. The acetylation status of mitochondrial proteins is regulated both by the level of acetyl-CoA and mitochondrial deacetylases of the sirtuin family, SIRT3 and SIRT5 [23]. SIRT4 is also located in mitochondria but shows only a minor deacetylase activity [24,25]. These sirtuins, as their nuclear (SIRT1, SIRT6, SIRT7) and cytosolic (SIRT2) homologs, are highly conserved NAD^+^-dependent proteins [26,27]. Both SIRT1 and SIRT3 have been shown to regulate heart metabolism [23]. SIRT1 can directly bind to and deacetylate peroxisome proliferator-activated receptor gamma coactivator-1 alpha (PGC-1 alpha), the master regulator of the mitochondrial biosynthesis [23,28]. The deacetylated form of PGC-1 alpha is capable of co-activating PPAR alpha in the transcriptional control of the FAO genes [29,30]. In addition, PGC-1 alpha has been shown to stimulate *Sirt3* gene expression in primary mouse hepatocytes and muscle cells [31,32]. The complex and interacting regulatory network of sirtuins, PPAR alpha, and PGC-1 is necessary for an efficient response to alterations in the levels of NAD^+^ and acetyl-CoA, the sensors of cellular metabolic state [33].

The goal of the present study was to investigate the role of mitochondrial acetylation in the regulation of cardiac injury after chronic radiation exposure. For this purpose, we studied radiation-induced alterations in the mitochondrial proteome and acetylome of ApoE ^-/-^ mice after 300 days of continuous low-dose rate (20 mGy/day) total body exposure to 137 Cs gamma rays. Thus, the irradiated mice received a cumulative dose of 6.0 Gy whilst the control mice were sham-irradiated. The ApoE ^-/-^ mice were used in this study since they are a well-established model in cardiovascular research [34,35,36]. Radiation-induced alterations of the FAO enzymes are very similar but more dominant in the ApoE ^-/-^ mice compared to the wild type [16].

## 2. Results

### 2.1. The Cardiac Mitochondrial Proteome Is Altered after Chronic Irradiation

Changes in the cardiac mitochondrial proteome of chronically irradiated mice were analyzed with label-free quantitative proteomics. A total number of 788 mitochondrial proteins were identified and quantified, of which 512 proteins were quantified at least with two unique peptides (2-UP) (Table S1). Among all 2-UP-identified proteins, 311 (61%) have been previously annotated as mitochondrial proteins based on MitoCarta 2.0 [37] (Table S1).

**Figure 1:**
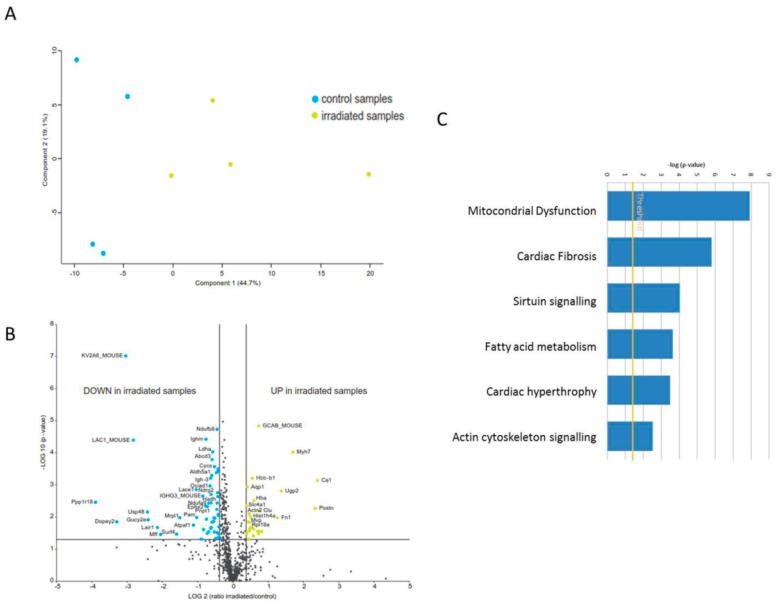
Proteome analysis of mitochondrial proteins in the irradiated heart. (**A**) Principal component analysis (PCA) based on all proteomic features. (**B**) Graphical representation of quantitative proteomics data of cardiac mitochondria after chronically exposure to accumulated doses of 6 Gy. Proteins are ranked in a volcano plot according to the −log10 of their statistical *p*-value (*y*-axis) and log2 fold change (*x*-axis). The yellow points represent the significantly more abundant proteins in cardiac mitochondria after irradiation, the blue points represent the significantly less abundant proteins. (**C**) The most significant canonical pathways altered by irradiation. The analyses were generated through the use of IPA (QIAGEN Inc., https://www.qiagenbio-informatics.com/products/ingenuity-pathway-analysis). Bars indicate canonical pathways and the *y*-axis displays the −(log p) enrichment significance. Taller bars are more significant than shorter bars.

To investigate differences in the proteome profiles between irradiated and control heart mitochondria, a principal component analysis (PCA) was performed based on all proteome features. Control and irradiated samples clustered into two separate groups (Figure 1A). The expression of 61 proteins was significantly different (2-UP; ± 1.3-fold; and ANOVA *p* < 0.05); of these, 41 proteins were down-regulated and 20 up-regulated in the irradiated samples (Figure 1B, Table S2).

A detailed analysis of the functional interactions and biological pathways was performed using Ingenuity Pathway Analysis (IPA) software. Mitochondrial dysfunction, cardiac fibrosis, sirtuin signalling, fatty acid metabolism, cardiac hypertrophy, and actin cytoskeleton signalling were the most affected pathways in the irradiated mitochondria compared to the control group (Figure 1C). PGC-1 alpha and PPAR alpha were predicted to be inhibited whilst transforming growth factor beta-1 (TGFB1) and p38 mitogen-activated protein kinases (P38MAPK) were predicted to be induced (Table S3). Differentially expressed proteins were associated with the cardiotoxicity related pathways such as cardiac enlargement, cardiac dysfunction, cardiac damage, and cardiac fibrosis (Table S3).

### 2.2. Long-Term Chronic Irradiation Causes Mitochondrial Protein Hyperacetylation

**Figure 2:**
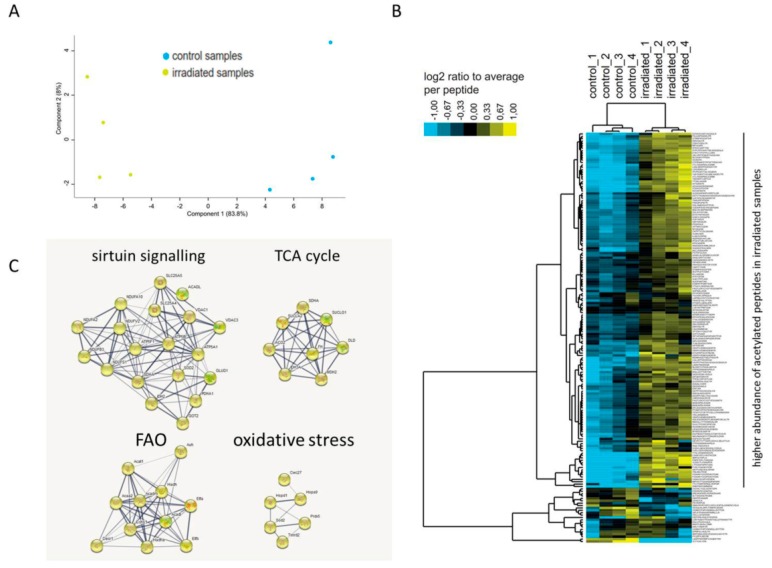
Protein-protein interaction analysis of acetylated proteins changed following total body irradiation. Principal component analysis (PCA) based on all acetylated peptides features (**A**). Heat map showing higher abundance of acetylated peptides (in yellow) in irradiated samples compared to the controls (**B**). Protein–protein interactions are analyzed by the STRING software tool (http://string-db.org) indicating the most affected protein clusters (**C**).

The acetylated proteins from control and irradiated mouse heart mitochondria were enriched as described in Materials and Methods. Of the 397 identified peptides in the enriched fractions, 172 peptides (43%) were found to be acetylated (Table S4). Among these peptides, the acetylation status of 142 unique peptides was significantly different (ANOVA *p* < 0.05) compared to the controls (Table S4). The irradiated mitochondria were clearly different from the controls based on the acetylation status of the peptides (Figure 2A). The irradiated mitochondria showed a generally higher abundance of acetylated peptides compared to the controls (Figure 2B).

The unique acetylated peptides were allocated to 71 acetylated proteins (Table 1). Of these, 49 possessed one unique acetylation site, whilst 22 had multiple acetylation sites. The acetylation status of 62 proteins was increased, whereas only three proteins showed hypoacetylation (Table 1 and Table S5). Aconitate hydratase (ACO2), dihydrolipoyl dehydrogenase (DLD), aspartate aminotransferase (GOT2), myosin-6 (MYH6) and ADP/ATP translocase 1 (SLC25A4) had peptides showing both increased and decreased acetylation levels (Table S5). The acetylated proteins showed no expression changes in the total proteome in response to irradiation, except in the case of somatic cytochrome C (CYCS), 2,4-dienoyl CoA reductase 1 (DECR1), dihydrolipoamide dehydrogenase (DLD), hydroxyacyl-coenzyme A dehydrogenase (HADH), and alpha subunit of succinate-CoA ligase (SUCLG1). These showed a slight reduction in protein expression (Table S5). Among the hyperacetylated proteins, there were several subunits of the mitochondrial respiratory chain as well as metabolic enzymes including Acyl-Coenzyme A dehydrogenase (ACADL), and trifunctional enzyme subunit alpha (HADHA) catalysing the first and last steps of FAO in mitochondria (Table 1 and Table S5). In general, the acetylated proteins were clustered in four main functional pathways: sirtuin signalling, tricarboxylic acid (TCA) cycle, FAO, and oxidative stress (Table 1 and Figure 2C).

### 2.3. Chronic Irradiation Decreases the Activity of Respiratory Complexes I and III and Cardiac ATP Level

**Figure 3:**
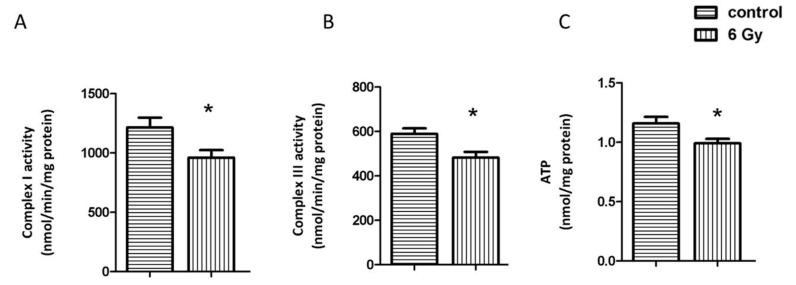
Analysis of the mitochondrial complex I and II activity, and cardiac ATP level. The activities of complex I and III were compared in irradiated and control samples (**A**,**B**). The ATP levels were compared in irradiated and control samples (**C**). The error bars represent standard error of the mean (± SEM) (t-test; * *p* < 0.05; *n* = 4).

Since several subunits of the mitochondrial electron transport chain (Complex I, Complex III, ATP synthase) were hyperacetylated in the irradiated samples (Table 1 and Table S5), we measured the activity of Complex I and Complex III. Both were significantly reduced in the irradiated samples (Figure 3A,B). The ATP level was also significantly reduced after chronic irradiation (Figure 3C). The increased acetylation status was presumably contributing to the reduced activity as the level of the complex proteins was only slightly or not significantly reduced (Tables S2 and S5).

### 2.4. Acetylation Impairs the Oxidative Stress Response in Irradiated Mitochondria

**Figure 4:**
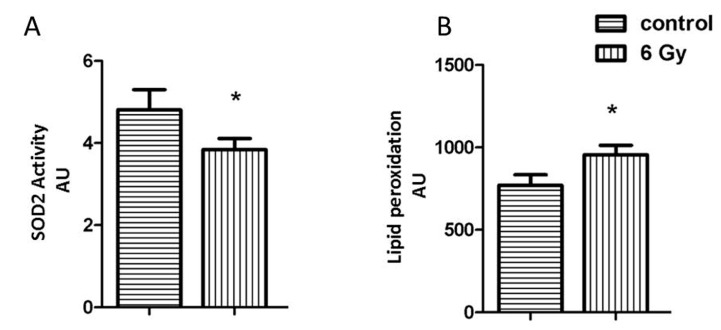
Analysis of the mitochondrial oxidative stress response. The expression level of mitochondrial SOD2 (**A**) and the amount of lipid peroxidation (**B**) as a marker of oxidative stress was measured in irradiated and control samples. The error bars represent standard error of the mean (± SEM) (t-test; * *p* < 0.05; *n* = 4).

Impairment of the mitochondrial complexes is involved in the enhanced oxidative stress [38]. The mitochondrial superoxide dismutase 2 (SOD2) is important in the clearance of mitochondrial reactive oxygen species. The acetylation level of SOD2 was increased, whilst the level of the total protein was not significantly changed in irradiated mitochondria (Tables S2 and S5). The activity of SOD2 showed a significant reduction in irradiated mitochondria, coinciding with the hyperacetylation (Figure 4A).

The effect of chronic radiation on the oxidative stress response was further studied by analysing the levels of malondialdehyde modification (lipid peroxidation marker) in mitochondrial samples. The level of malondialdehyde-modified proteins was significantly increased after irradiation (Figure 4B).

### 2.5. Mitochondrial NAD^+^/NADH Level Is Reduced after Chronic Irradiation

**Figure 5:**
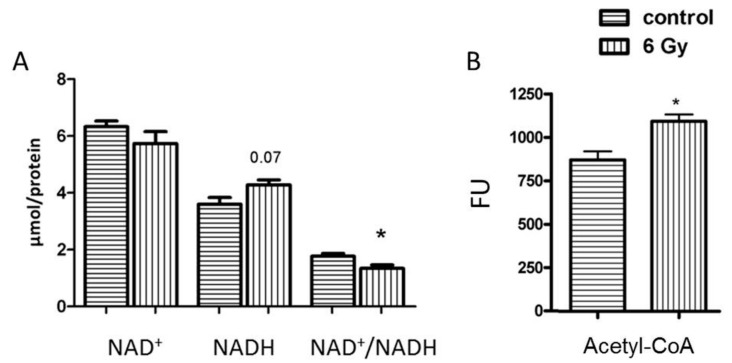
Analysis of the mitochondrial NAD^+^, NADH and NAD^+^/NADH and Acetyl-CoA. The concentration of NAD^+^, NADH and NAD^+^/NADH (**A**) and Acetyl-CoA (**B**) was compared in samples from irradiated and control groups. The error bars represent standard error of the mean (± SEM) (t-test; * *p* < 0.05; *n* = 4).

The deficiency of mitochondrial complex I results in alteration of NAD^+^ homeostasis and subsequently alters the protein acetylation status [39]. The NAD^+^/NADH ratio in irradiated mitochondria decreased compared to controls (Figure 5A).

### 2.6. The Level of Acetyl-CoA Is Enhanced in Irradiated Mitochondria

Protein acetylation is partly mediated by non-enzymatic mechanism mainly controlled by acetyl-CoA level in mitochondria [22]. In accordance with the radiation-induced hyperacetylation, the analysis of acetyl-CoA amounts showed a significantly enhanced level of acetyl-CoA in irradiated mitochondria compared to that of the controls (Figure 5B).

### 2.7. Irradiation Negatively Influences the Activity and Expression of Sirtuins

**Figure 6:**
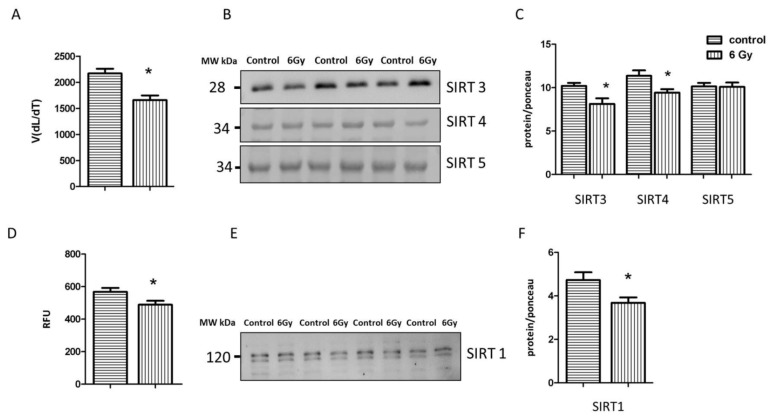
Analysis of the sirtuin proteinsactivity and expression. The activity of sirtuin proteins was measured in mitochondria fraction using pan-SIRT assay. The error bars represent standard error of the mean (± SEM) (t-test; * *p* < 0.05; *n* = 3) (**A**). Immunoblotting analysis of SIRT3, SIRT4, SIRT5 was performed in the mitochondrial lysates from each group (**B**, **C**). The columns represent the average ratios of relative protein expression in control and irradiated samples after background correction (t-test; * *p* < 0.05; *n* = 3) (**C**). The activity of SIRT1 was compared between irradiated and control samples (t-test; * *p* < 0.05; *n* = 4) (**D**). Immunoblotting analysis of SIRT1 was performed using whole heart lysate. The error bars represent standard error of the mean (t-test; * *p* < 0.05; *n* = 4) (**E**,**F**). The amount of the total protein was measured by Ponceau S staining for an accurate comparison between the groups. The error bars represent standard error of the mean (± SEM) (t-test; * *p* < 0.05; *n* = 3).

Since sirtuins are NAD^+^-dependent deacetylases, the radiation-associated decrease in the NAD^+^/NADH ratio may affect their activity and expression and lead to hyperacetylation. The analysis indicated a significantly decreased level of total mitochondrial sirtuin activity in the irradiated samples (Figure 6A). The immunoblot analysis of the level of different mitochondrial sirtuins (SIRT3, SIRT4, SIRT5) showed that the expression of SIRT3 and SIRT4 was significantly reduced in irradiated mitochondria in comparison to controls (Figure 6B,C and Figure S1A–E), whereas the level of SIRT5 remained unchanged.

The activity and expression of SIRT1 were tested in the corresponding control and irradiated heart tissue. Both were significantly reduced in the irradiated heart (Figure 6D–F and Figure S2A,B).

### 2.8. Reduced Sirtuin Activities Contribute to Cardiac Senescence

**Figure 7:**
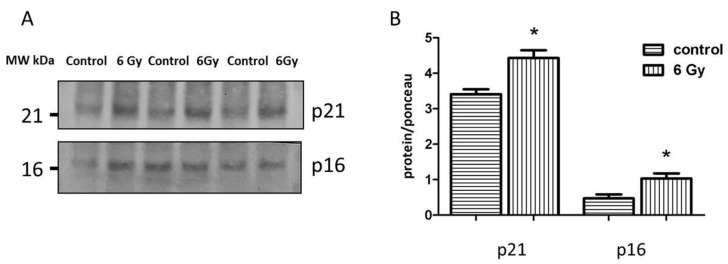
Analysis of the senescence-associated proteins. Immunoblotting analysis of the senescence markers (p21 and p16) in whole heart lysate (**A**,**B**). The amount of the total protein was measured by Ponceau S staining for an accurate comparison between the groups. The error bars represent standard error of the mean (± SEM) (t-test; * *p* < 0.05; *n* = 3).

To investigate the evidence of accumulated senescent cells in irradiated cardiac tissue, the expression levels of senescence-associated proteins p21 and p16 were analyzed in the heart lysate using immunoblotting. The levels of p21 and p16 were significantly increased in the irradiated samples compared to the controls (Figure 7A,B and Figure S3A–C), suggesting the early onset of cardiac ageing.

### 2.9. Mitochondrial Acetylation Is Involved in Radiation-Induced Cardiac Metabolism Alteration

**Figure 8:**
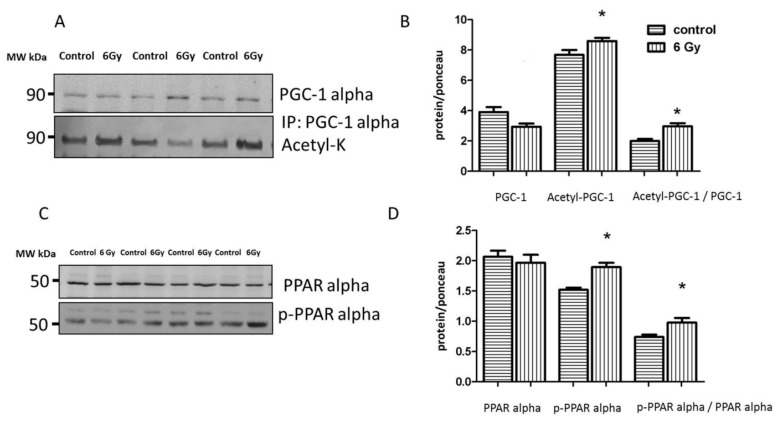
Analysis of the cardiac PGC-1 and PPAR alpha protein expression. Immunoblot analysis of total PGC1, and acetylated form (inactive) in whole heart lysate is shown (**A**). The columns represent the average ratios of relative protein expression in control and irradiated samples. The error bars represent standard error of the mean (± SEM) (t-test; * *p* < 0.05; *n* = 3) (**B**). Immunoblot analysis of total and phospho-PPAR alpha (Ser12) in whole heart lysate samples is shown (**C**). The amount of the total protein was measured by Ponceau S staining for an accurate comparison between the groups. The columns represent the average ratios of relative protein expression in control and irradiated samples after background correction (**D**). The error bars represent standard error of the mean (± SEM) (t-test; * *p* < 0.05; *n* = 4).

The proteome and acetylome analysis of the irradiated mitochondria showed alteration of a cluster of metabolic proteins regulated by PGC-1 alpha and PPAR alpha (Tables S2 and S3). Based on IPA upstream regulator analysis of the mitochondrial proteome, PPAR alpha and its coactivator PGC-1 alpha were predicted to be inhibited following irradiation (Table S3). Since PPAR alpha and PGC-1 alpha are not mitochondrial proteins, we analyzed the expression levels of these factors in the whole heart lysate. PGC-1 alpha activity is regulated by acetylation [40]. The results confirmed the significantly enhanced level of acetylated (inactive) PGC-1 alpha in irradiated heart tissue (Figure 8A,B and Figure S4A–C). The activity of PPAR alpha in the heart is regulated by the phosphorylation of Ser12, increased phosphorylation meaning deactivation [41]. The ratio of phosphorylated (inactive) to total protein was significantly increased in the irradiated heart samples compared to the control group (Figure 8C,D and Figure S2A,C,D), indicating a radiation-related reduction of PPAR alpha transcriptional activity in the chronically irradiated heart. This was in agreement with its predicted inactivation based on the changes in the mitochondrial proteome (Table S3).

## 3. Discussion

The goal of the present study was to investigate the late cardiac effects of low-dose-rate chronic exposure. This is important as several recent studies have suggested an excess radiation-induced risk of heart disease at low occupational and environmental dose rate levels [1]. For example, Mayak nuclear workers show an increased risk for ischemic heart disease that was shown to increase linearly in relation to the total external gamma-ray dose in both male and female workers [8,9].

Our previous data using cardiac left ventricle autopsies of male Mayak workers showed a dose-dependent increase in the number of dysregulated proteins [18,42]. In all dose groups, the most affected pathway was cardiac energy production [18]. Especially influenced were mitochondrial proteins, particularly members of the respiratory chain complexes I and III [18]. Here, we report a study using a mouse model that identifies the protein acetylation as a potential contributor mechanism in radiation-induced mitochondrial metabolic disruption. To our best knowledge, it is the first study to investigate the effect of chronic ionizing radiation on the cardiac mitochondrial acetylome and proteome. The study correlates the omics findings with functional analysis to address the molecular mechanism involved in the late cardiac effect of irradiation.

Cardiac mitochondrial proteins are the known targets of post-translational acetylation [22]. The acetylation status of proteins is mainly regulated by a NAD^+^-dependent deacetylases known as sirtuins [43]. Among the mitochondrial sirtuins, SIRT3 is a major regulator of the mitochondrial acetylation status [43]. More than 60% of mitochondrial proteins contain acetylation sites [22], and the majority of acetylated mitochondrial proteins are involved in energy metabolism [44], including the aforementioned oxidative phosphorylation but also FAO, and the TCA cycle [45,46,47].

The present study clearly shows a radiation-induced increase in the acetylation status of several subunits of mitochondrial respiratory complexes I and III that coincides with their decreased activity. It has been shown previously that deficiencies in oxidative phosphorylation, in particular in Complex I, lead to a decrease in NAD^+^/NADH ratio and inhibition of SIRT3 activity in the heart [39]. In accordance with this, we also observe a reduced level of NAD^+^/NADH and decreased expression and activity of SIRT3 in the irradiated mitochondria.

Proteome and acetylome analyses of this study show that it is mainly mitochondrial metabolic enzymes that are affected after irradiation. SIRT3 null mice have been shown to have a 33% lower FAO in the heart compared to wild type [20]. The first enzyme of FAO, ACADL, is a known target of SIRT3 [22]. Hyperacetylation of ACADL, as observed in the irradiated mitochondria in this study, is reported in different heart diseases such as cardiac hypertrophy [48] and heart failure [49]. We have shown previously that local heart irradiation (2 Gy X-ray) results in down-regulation of enzymes involved in long-chain fatty acid degradation, including ACADL, 40 weeks after the radiation exposure in male ApoE ^-/-^ mice [16]. Accumulation of long-chain fatty acids is a typical feature of aged cardiac mitochondria [50]. In accordance with this, mice lacking SIRT3 show similar cardiac pathologies as the aged wild-type mice such as cardiac hypertrophy and fibrosis [51,52].

The sirtuin family is known to play a role in cardiac ageing [53,54,55,56]. Our analysis confirms radiation-responsive up-regulation of p21 and p16 in cardiac tissue suggesting accumulated senescent cells in heart tissue following irradiation. Accumulation of senescent cells in aged tissues impairs tissue function and affects the neighbouring cells via senescence-associated secretory phenotype (SASP) [57]. Increased levels of p21 and p16 have been shown previously in the aged heart [58,59].

SIRT3 plays an essential role in the regulation of mitochondrial redox state [22], since it is able to deacetylate SOD2 and induce its activity [60,61] leading to changes in the reactive oxygen species (ROS) level [61]. The present study shows that chronic irradiation results in increased acetylation of SOD2 and reduction of its activity. In good agreement with this, irradiated mitochondria show here enhanced level of lipid peroxidation, a biomarker of oxidative stress [62]. We have shown previously that local acute heart irradiation permanently increases mitochondrial ROS production and this is associated with enhanced protein oxidation in cardiac mitochondria [16,17]. Similarly, the proteome analysis of the heart of Mayak workers demonstrated a dose-dependent reduction of nuclear factor erythroid 2 (NFE2)-related factor 2 (Nrf2), key regulator of oxidative stress response pathway and several of its target proteins including catalase, superoxide dismutases (SOD1 and SOD2), peroxiredoxins (1, 2, 3, 5, and 6), and glutathione-S-transferases (kappa1, mu2, mu3, omega 1, pi1) and enhanced protein carbonylation in the highest dose group (< 500 mGy) [18].

These findings suggest that cardiac mitochondria are both the source and target of ROS after radiation exposure, independent of the dose rate. A comparison of our previous studies using male mice with the data presented here for female animals indicates that irradiation does not appear to cause a different mitochondrial response in males and females [16,17]. We have previously shown the downregulation of several metabolic enzymes in male human and male animal heart proteome following irradiation [15,16,18]. This event is accompanied by inactivation of cardiac metabolic transcription factor PPAR alpha [15,18]. Here, in the female mouse hearts, we also see the inactivation of PPAR alpha after irradiation, suggesting that radiation-induced impairment of cardiac energy metabolism might be a signature of radiation-induced cardiac pathology independent of gender. It remains to be determined whether mitochondrial acetylation differs between males and females.

The level of SIRT3 is not only dependent on the availability of NAD^+^. SIRT3 expression is reported to be regulated by SIRT1 either via the AMPK-PGC1 pathway or by deacetylation [63,64]. We observed here reduction in expression and activity of both sirtuins in irradiated hearts. The role of SIRT1 and SIRT3 in the maintaining of cellular metabolism and response to stress has been previously shown [63,65].

One of the transcriptional controllers of the *Sirt3* gene is PGC-1 alpha, a coactivator of PPAR alpha transcription complex [31,32]. Interestingly, PGC-1 alpha is deacetylated and thereby activated as a cofactor by SIRT1 [40] that, in its turn, results in the transcription of genes involved in oxidative phosphorylation and FAO [40,66]. Furthermore, SIRT1 has been shown to bind PPAR alpha directly and facilitates PGC-1/ PPAR alpha interactions [67]. We observed here a marked reduction of the expression and activity of both sirtuins that was accompanied by the inactivation of PGC-1 alpha and PPAR alpha.

**Figure 9:**
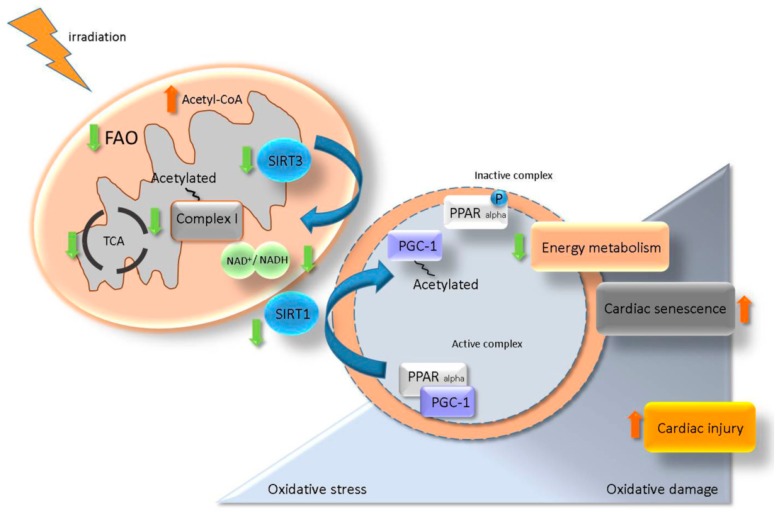
A proposed model for the role of SIRT/ PGC-1/ PPAR alpha network in regulation of radiation-induced cardiac injury. Irradiation impairs the mitochondrial complex I activity resulting in NAD^+^ homeostasis alteration. A change in the level of NAD^+^/NADH reduces the activity of SIRT3 and enhances the acetylation state of mitochondrial proteins. NAD^+^/NADH alteration also affects the SIRT1 activity and impairs the PGC-1/ PPAR alpha transcription complex via an increased level of acetylated (inactive) PGC-1. Deactivation of PGC-1/ PPAR alpha is associated with a low level of myocardial metabolism, elevated oxidative damage and accelerated senescence contributing to the radiation-induced cardiac injury.

Data presented here highlight the role of the SIRT/ PGC-1/ PPAR alpha network in the maintenance of cardiac metabolism [40,63,64]. A putative model for the role of SIRT/ PGC-1/ PPAR alpha regulatory axis in the radiation-induced cardiac injury is suggested in Figure 9. It proposes that irradiation caused mitochondrial metabolic impairment, enhanced oxidative damage and accelerated senescence.

## 4. Materials and Methods

### 4.1. Animals

ApoE ^-/-^ mice on the C57BL/6J background (Charles River Laboratories, Wilmington, Massachusetts, USA) were bred at the IES animal facility under specific pathogen-free (SPF) conditions. All experiments were conducted according to the legal regulations in Japan and the Guidelines for Proper Conduct of Animal Experiments (2006, Science Council of Japan, Cabinet Office) (http://www.scj.go.jp/ja/info/kohyo/pdf/kohyo-20-k16-2e.pdf). The experiment protocol (Project ID Codes 24-20 (March 19, 2013), 24-21 (March 19, 2013) and 25-16 (March 27, 2014)) was reviewed and approved by the Institutional Animal Care and Use Committee (IACUC) of the Institute for Environmental Sciences according to the science-based guidelines for Laboratory Animal Care (https://www.ncbi.nlm.nih.gov/books/NBK25422/). The mice were fed with standard rodent chow ad libitum and maintained at a 12 h light/dark cycle.

### 4.2. Irradiation

At 8 weeks of age, female mice (16 mice, 8 for heart analysis and 8 for mitochondria analysis) were subjected to chronic irradiation with gamma rays for 300 days using a 137 Cs source (22 hr/day; dose rate 20 mGy/day) reaching cumulative total body doses of 6.0 Gy. Controls were sham-irradiated. Mice were euthanized immediately after the radiation exposure using CO_2_ asphyxiation. The animals exposed to a total dose of 6 Gy appeared apparently healthy until the termination of the study. No neoplasms were detected in all examined animals. There was no significant difference in the body weight, heart weight and heart/body weight ratio of the irradiated mice compared to the non-irradiated mice (Table S6).

### 4.3. Cardiac Mitochondria Isolation

Cardiac mitochondria were isolated using differential centrifugation and discontinuous Percoll gradient technique as described before [17]. To investigate the mitochondrial purity, mitochondrial marker voltage-dependent anion-selective channel protein (VDAC) and endoplasmic reticulum marker, binding immunoglobulin protein (BiP) were measured using immunoblotting in the mitochondrial and heart tissue lysates. VDAC was enriched in the mitochondrial fraction compared to the whole heart tissue, whilst endoplasmic reticulum marker BiP was strongly reduced (Figure S5A–C).

### 4.4. Proteome Analysis

Mitochondrial proteins from control and irradiated hearts (four mice per group) were quantified using label-free LC-MS/MS analysis as described before [18]. Briefly, samples were analyzed on the LTQ-OrbitrapXL (Thermo Scientific, Wilmington, Massachusetts, USA) online coupled to the Ultimate 3000 RSPL (Thermo) equipped with a reversed-phase analytical column (Thermo Scientific Acclaim PepMap 100 C18, 3µm, 250mm length, 0.075mm I.D.). Label-free quantifications were performed using the Progenesis QI for proteomics software (Waters) in combination with the search engine Mascot (Matrix Science) and the Swiss-Prot mouse database (16872 sequences). For final quantifications, proteins were identified with at least 2 unique peptides and with ratios greater than 1.30-fold or less than 0.77-fold (ANOVA; *p* < 0.05) were defined as being significantly differentially expressed.

### 4.5. Acetylome Analysis

Protein extracts (300 μg) of mitochondria isolated from control and irradiated hearts (four mice per group) were resolved in 1% Triton, 100 mM Tris buffer, pH 7.6, and were subjected to overnight in-solution tryptic digestion. Acetylated peptides were enriched from lysates using agarose-conjugated anti-acetyl lysine (#ICP0388, ImmunChem, Burnaby, Canada) and eluted using 0.1% TFA as described previously [68]. Acetylated peptides were quantified similarly to the proteome analysis described above. The acetylation of lysine and N-terminus of protein was selected as variable modifications in addition to standard search settings.

For quantification, all unique peptides with *p* < 0.05 (resulting peptide FDR 3.04%) for each identified protein were included. No minimal thresholds were set for the method of peak picking or selection of data used for quantification. Peptides showing a significant difference (*p* < 0.05) between the two groups were considered to be significantly changed in their acetylation status.

To control the effect of proteins changes in the irradiated mitochondria that might contribute to altered levels of acetylated peptides, the change in the acetylated state of each peptide was normalized by dividing the ratio (irradiated /control) of the acetylated peptide to the ratio (irradiated /control) of the corresponding protein obtained from proteome quantification.

### 4.6. Principal Components Analysis

Principal components analysis was performed in the Perseus software [69] based on the log2 of the protein/peptide abundance ratios for every single sample to the average protein/peptide abundance in all samples.

### 4.7. Heatmap Generation

A heatmap of the acetylome data was generated using the log2 of the peptide abundance ratios for every single sample to the average peptide abundance in all samples, with peptides with an abundance above average given in yellow and lower proteins in blue. Ratios were used for complete hierarchical clustering based on euclidean distance using Cluster 3.0 software (Stanford, CA, USA) [70].

The resulting tree and heatmap were visualised with Java Treeview (http://www.eisenlab.org/index.html?page_id=42).

### 4.8. Protein-Protein Interaction and Signalling Network

The analyses of protein–protein interactions and signalling networks were performed by the software tools STRING 11 (http://string-db.org) [71] and INGENUITY Pathway Analysis (IPA) (QIAGEN Inc., https://www.qiagenbio-informatics.com/products/ingenuity-pathway-analysis) [72].

### 4.9. Sirtuin Activity Assay

The mitochondrial global sirtuin activity was measured by SIRT-Glo™ Assay (#G6450; Promega, Madison, Wisconsin, USA) as recommended by the manufacturer. The activity of SIRT1 was measured in whole heart lysate using fluorometric assay (ab156065; Abcam, Cambridge, UK) according to the manufacturer´s recommendations.

### 4.10. Complex I and Complex III Activity

Complex I and Complex III activities were measured in isolated mitochondria using the assay kit (#700930; MitoCheck®; USA and #K520; BioVision, Milpitas, CA, USA) according to the manufacturer’s recommendations.

### 4.11. ATP Assay

The ATP levels were measured in the frozen heart samples using a commercially available kit (ab83355, Abcam; USA) according to the manufacturer’s instructions.

### 4.12. Acetyl-CoA Assay

Mitochondrial Acetyl-CoA levels were quantified with a fluorometric assay kit (#K317; BioVision; USA) according to the manufacturer’s instructions.

### 4.13. NAD^+^/ NADH Assay

Mitochondrial NAD^+^/NADH levels were quantified with a commercially available kit (MAK037, Sigma Chemical, St. Louis, Missouri, USA) according to the manufacturer’s instructions.

### 4.14. SOD2 Activity Assay

Superoxide dismutase activity was measured in isolated mitochondria by colourimetric assay (ab65354, Abcam; USA) as recommended by the manufacturer.

### 4.15. Lipid Peroxidation Assay

Lipid peroxidation was measured in isolated mitochondria using the colourimetric assay kit (ab118970; Abcam; USA) according to the manufacturer´s instructions.

### 4.16. Immunoblot Analysis

Mitochondrial protein lysate (10 µg) or heart tissue lysate (10 µg) were separated by gradient 4-12% SDS-PAGE were transferred to nitrocellulose membranes (GE Healthcare; USA) using a Trans-Blot Turbo™ system (Bio-Rad, Hercules, California, USA) according to manufacturer´s recommendations. The membranes were blocked using 3% BSA in TBS, pH 7.4, for 1 h at room temperature, washed three times in 10 mM Tris-HCl, pH 7.4, 150 mM NaCl for 5 min and incubated overnight at 4 °C with primary antibodies using dilutions recommended by the manufacturer. Immunoblot analysis was performed using anti-SIRT1 (#9475S; Cell Signaling Technology, Danvers, Massachusetts, USA), anti-SIRT3 (#5490; Cell Signaling Technology), anti-SIRT4 (sc-135797; Santa Cruz Biotechnology; USA), anti-SIRT5 (sc-271635; Santa Cruz Biotechnology, Dallas, Texas, USA), anti-PGC-1 (ab54481; Abcam; USA), anti-PPAR alpha (sc-9000; Santa Cruz Biotechnology; USA), anti-phospho-PPAR alpha, S-12 (ab3484; Abcam; USA), anti-acetylated lysine (#9441; Cell Signaling Technology; USA), anti-VDAC (#4661; Cell Signaling Technology; USA), anti-BiP (#3177; Cell Signaling Technology; USA), anti-p21 (#2947; Cell Signaling Technology; USA) and anti-p16 (#80772; Cell Signaling Technology; USA). After washing three times, the blots were incubated with the appropriate horseradish peroxidase-conjugated or alkaline phosphatase-conjugated anti-mouse, anti-rabbit or anti-goat secondary antibody (Santa Cruz Biotechnology) for 2 h at room temperature and developed using the ECL system (GE Healthcare, Chicago, Illinois, USA) or 1-stepTM NBT/BCIP method (ThermoFisher, Wilmington, Massachusetts, USA) following standard procedures. Reversible Ponceau staining was used as the loading control. For immunoprecipitation, 100 µg of protein lysate was incubated overnight with control IgG or PGC-1 antibody; the mixed reaction was precipitated with Protein A/G Agarose beads (Santa Cruz Biotechnology; USA) according to the manufacturer’s instructions and immunoprecipitated proteins were analyzed by immunoblotting.

Full-length images of Ponceau S staining and antibody detection of the replicates that not shown in the main results figures are provided in supplementary materials (Supplementary Figures S1–S5).

### 4.17. Statistical Analysis

Comparative analysis of the data was carried out using the Student´s t-test (unpaired). The significance levels were p* < 0.05.

### 4.18. Data Availability

The raw MS data have been deposited in the RBstore database, Study ID: 1151: (https://www.storedb.org/store_v3/study.jsp?studyId=1151).

## 5. Conclusions

This study emphasizes the role of acetylation in the mitochondrial response to chronic irradiation and suggests a strong interdependence between mitochondrial acetylation and energy production. The impaired cardiac metabolism, the increased ROS levels and especially the reduced expression of SIRT1 and SIRT3, the longevity proteins, are all reminiscent of the processes seen in the ageing heart. These and other data [3] indicate that radiation-induced accelerated ageing may play a role in cardiac injury seen in occupationally exposed populations.

## Figures and Tables

**Table 1:** List of significantly changed acetylated mitochondrial proteins following irradiation (*p* < 0.05). The number of acetylated peptides, Gene Ontology (GO) description and GO Accession are shown for each acetylated protein.

No.	ID	Description	No. Of Hyperacetylated Peptides	No. Of Hypoacetylated Peptides	GO Description	GO Accession
1	ACAA2	3-ketoacyl-CoA thiolase	2	0	FAO	GO:0006635
2	ACADL	Long-chain specific acyl-CoA dehydrogenase	2	0	FAO	GO:0006635
3	ACADM	Medium-chain specific acyl-CoA dehydrogenase	1	0	FAO	GO:0006635
4	ACAT1	Acetyl-CoA acetyltransferase	1	0	FAO	GO:0006635
5	ACO2	Aconitate hydratase	6	1	TCA metabolic process	GO:0072350
6	ACOT13	Acyl-coenzyme A thioesterase 13	4	0	acyl-CoA hydrolase activity	GO:0047617
7	ACOT5	Acyl-coenzyme A thioesterase 5	1	0	acyl-CoA hydrolase activity	GO:0047617
8	ALDH4A1	Delta-1-pyrroline-5-carboxylate dehydrogenase	1	0	TCA metabolic process	GO:0072350
9	ATP5A1	ATP synthase subunit alpha	1	0	ATP metabolic process	GO:0046034
10	ATP5B	ATP synthase subunit beta	2	0	ATP metabolic process	GO:0046034
11	ATP5F1	ATP synthase F(0) complex subunit B1	3	0	ATP metabolic process	GO:0046034
12	ATP5H	ATP synthase subunit d	9	0	ATP metabolic process	GO:0046034
13	ATP5L	ATP synthase subunit g	2	0	ATP metabolic process	GO:0046034
14	ATP5O	ATP synthase subunit O	1	0	ATP metabolic process	GO:0046034
15	AUH	Methylglutaconyl-CoA hydratase	1	0	FAO	GO:0006635
16	CBLB	E3 ubiquitin-protein ligase	1	0	ubiquitin protein ligase binding	GO:0031625
17	COX4I1	Cytochrome c oxidase subunit 4 isoform 1	2	0	ATP metabolic process	GO:0046034
18	CUX1	Homeobox protein cut-like 1	1	0	DNA binding	GO:0003677
19	CWC27	Peptidyl-prolyl cis-trans isomerase	1	0	protein folding	GO:0006457
20	CYCS	Cytochrome c	1	0	ATP metabolic process	GO:0046034
21	DECR1	2-4-dienoyl-CoA reductase	1	0	FAO	GO:0006635
22	DLD	Dihydrolipoyl dehydrogenase	3	1	ATP metabolic process	GO:0046034
23	DLST	Dihydrolipoyllysine-residue succinyltransferase component of 2-oxoglutarate dehydrogenase complex	1	0	TCA metabolic process	GO:0072350
24	ECHS1	Enoyl-CoA hydratase	1	0	FAO	GO:0006635
25	ETFA	Electron transfer flavoprotein subunit alpha	3	1	FAO	GO:0006635
26	ETFB	Electron transfer flavoprotein subunit beta	1	0	FAO	GO:0006635
27	FAM187A	Ig-like V-type domain-containing protein	1	0	enzyme binding	GO:0019899
28	FGD6	RhoGEF and PH domain-containing protein 6	1	0	regulation of GTPase activity	GO:0043087
29	FH	Fumarate hydratase	5	0	TCA metabolic process	GO:0072350
30	GBAS	Protein NipSnap homolog 2	1	0	ATP metabolic process	GO:0046034
31	GLUD1	Glutamate dehydrogenase 1	1	0	TCA metabolic process	GO:0072350
32	GOT2	Aspartate aminotransferase	3	1	amino acid binding	GO:0016597
33	HADH	Hydroxyacyl-coenzyme A dehydrogenase	1	0	FAO	GO:0006635
34	HADHA	Trifunctional enzyme subunit alpha	7	0	FAO	GO:0006635
35	HIST1H1C	Histone H1	1	0	DNA binding	GO:0003677
36	HIST1H1E	Histone H1	1	0	ATP binding	GO:0005524
37	HMGCL	Hydroxymethylglutaryl-CoA lyase	1	0	fatty-acyl-CoA binding	GO:0000062
38	HSPA9	Stress-70 protein	1	0	unfolded protein binding	GO:0051082
39	HSPD1	60 kDa heat shock protein	1	0	unfolded protein binding	GO:0051082
40	IDH2	Isocitrate dehydrogenase [NADP]	12	0	TCA metabolic process	GO:0072350
41	IDH3A	Isocitrate dehydrogenase [NADP]	0	1	TCA metabolic process	GO:0072350
42	LRRC40	Leucine-rich repeat-containing protein 40	1	0	cellular process	GO:0009987
43	MDH1B	Putative malate dehydrogenase 1B	1	0	TCA metabolic process	GO:0072350
44	MDH2	Malate dehydrogenase	6	0	TCA metabolic process	GO:0072350
45	MMEL1	Membrane metallo-endopeptidase-like 1	1	0	endopeptidase activity	GO:0004175
46	MRGPRA1	Mas-related G-protein coupled receptor member A1	0	1	G protein-coupled receptor activity	GO:0004930
47	MYH6	Myosin-6	2	1	actin-dependent ATPase activity	GO:0030898
48	NDUFA2	NADH dehydrogenase [ubiquinone] 1 alpha subcomplex subunit 2	1	0	NADH dehydrogenase (ubiquinone) activity	GO:0008137
49	NDUFAB1	Acyl carrier protein	0	1	acyl binding	GO:0000035
50	NDUFB3	NADH dehydrogenase [ubiquinone] 1 beta subcomplex subunit 3	1	0	NADH dehydrogenase (ubiquinone) activity	GO:0008137
51	NDUFS1	NADH-ubiquinone oxidoreductase 75 kDa subunit	2	0	NADH dehydrogenase (ubiquinone) activity	GO:0008137
52	NDUFV2	NADH dehydrogenase [ubiquinone] flavoprotein 2	1	0	NADH dehydrogenase (ubiquinone) activity	GO:0008137
53	NIT2	Omega-amidase NIT2	1	0	omega-amidase activity	GO:0050152
54	OXCT1	Succinyl-CoA:3-ketoacid coenzyme A transferase 1	1	0	3-oxoacid CoA-transferase activity	GO:0008260
55	PAPOLB	Poly(A) polymerase beta	1	0	RNA polymerase binding	GO:0070063
56	PDHA1	Pyruvate dehydrogenase E1 component subunit alpha	1	0	TCA metabolic process	GO:0072350
57	PRDX5	Peroxiredoxin-5	2	0	response to oxygen radical	GO:0000305
58	PROSC	Proline synthase co-transcribed bacterial homolog protein	1	0	pyridoxal phosphate binding	GO:0030170
59	RPIA	Ribose-5-phosphate isomerase	1	0	carbohydrate binding	GO:0030246
60	SDHA	Succinate dehydrogenase [ubiquinone] flavoprotein subunit	4	0	TCA metabolic process	GO:0072350
61	SLC25A3	Phosphate carrier protein	1	0	phosphate transmembrane transporter	GO:0005315
62	SLC25A4	ADP/ATP translocase 1	2	1	ATP:ADP antiporter activity	GO:0005471
63	SLC25A5	ADP/ATP translocase 2	1	0	ATP:ADP antiporter activity	GO:0005471
64	SOD2	Superoxide dismutase [Mn]	1	0	response to oxygen radical	GO:0000305
65	SUCLA2	Succinate--CoA ligase [ADP-forming] subunit beta	1	0	TCA metabolic process	GO:0072350
66	SUCLG1	Succinate--CoA ligase [ADP/GDP-forming] subunit alpha	1	0	TCA metabolic process	GO:0072350
67	TXNRD2	Thioredoxin reductase 2	1	0	response to oxygen radical	GO:0000305
68	UQCRB	Cytochrome b-c1 complex subunit 7	1	0	ATP metabolic process	GO:0046034
69	UQCRC1	Cytochrome b-c1 complex subunit 1	1	0	ATP metabolic process	GO:0046034
70	VDAC1	Voltage-dependent anion-selective channel protein 1	5	0	anion channel activity	GO:0005253
71	VDAC3	Voltage-dependent anion-selective channel protein 3	1	0	anion channel activity	GO:0005253

## Supplementary Materials

Supplementary materials can be found at https://www.mdpi.com/1422-0067/20/20/5239/s1.

## References

[1] Little M.P. (2016). Radiation and circulatory disease. Mutat. Res..

[2] Little M.P., Azizova T.V., Bazyka D., Bouffler S.D., Cardis E., Chekin S., Chumak V.V., Cucinotta F.A., de Vathaire F., Hall P. (2012). Systematic review and meta-analysis of circulatory disease from exposure to low-level ionizing radiation and estimates of potential population mortality risks. Environ. Health Perspect..

[3] Tapio S. (2016). Pathology and biology of radiation-induced cardiac disease. J. Rad. Res..

[4] Swerdlow A.J., Higgins C.D., Smith P., Cunningham D., Hancock B.W., Horwich A., Hoskin P.J., Lister A., Radford J.A., Rohatiner A.Z. (2007). Myocardial infarction mortality risk after treatment for Hodgkin disease: A collaborative British cohort study. J. Natl. Cancer Inst..

[5] Darby S.C., Ewertz M., McGale P., Bennet A.M., Blom-Goldman U., Bronnum D., Correa C., Cutter D., Gagliardi G., Gigante B. (2013). Risk of ischemic heart disease in women after radiotherapy for breast cancer. N. Engl. J. Med..

[6] Carr Z.A., Land C.E., Kleinerman R.A., Weinstock R.W., Stovall M., Griem M.L., Mabuchi K. (2005). Coronary heart disease after radiotherapy for peptic ulcer disease. Int. J. Radiat. Oncol. Biol. Phys..

[7] Tukenova M., Guibout C., Oberlin O., Doyon F., Mousannif A., Haddy N., Guerin S., Pacquement H., Aouba A., Hawkins M. (2010). Role of cancer treatment in long-term overall and cardiovascular mortality after childhood cancer. J. Clin. Oncol..

[8] Azizova T.V., Muirhead C.R., Moseeva M.B., Grigoryeva E.S., Vlasenko E.V., Hunter N., Haylock R.G., O’Hagan J.A. (2012). Ischemic heart disease in nuclear workers first employed at the Mayak PA in 1948-1972. Health Phys..

[9] Azizova T.V., Grigoryeva E.S., Haylock R.G., Pikulina M.V., Moseeva M.B. (2015). Ischaemic heart disease incidence and mortality in an extended cohort of Mayak workers first employed in 1948-1982. Br. J. Radiol..

[10] Azizova T.V., Muirhead C.R., Druzhinina M.B., Grigoryeva E.S., Vlasenko E.V., Sumina M.V., O’Hagan J.A., Zhang W., Haylock R.G., Hunter N. (2010). Cardiovascular diseases in the cohort of workers first employed at Mayak PA in 1948-1958. Radiat. Res..

[11] Gillies M., Richardson D.B., Cardis E., Daniels R.D., O’Hagan J.A., Haylock R., Laurier D., Leuraud K., Moissonnier M., Schubauer-Berigan M.K. (2017). Mortality from Circulatory Diseases and other Non-Cancer Outcomes among Nuclear Workers in France, the United Kingdom and the United States (INWORKS). Radiat. Res..

[12] Zhang W., Haylock R.G.E., Gillies M., Hunter N. (2019). Mortality from heart diseases following occupational radiation exposure: Analysis of the National Registry for Radiation Workers (NRRW) in the United Kingdom. J. Radiol. Prot..

[13] Ruhm W., Azizova T., Bouffler S., Cullings H.M., Grosche B., Little M.P., Shore R.S., Walsh L., Woloschak G.E. (2018). Typical doses and dose rates in studies pertinent to radiation risk inference at low doses and low dose rates. J. Radiat. Res..

[14] Hunter N., Kuznetsova I.S., Labutina E.V., Harrison J.D. (2013). Solid cancer incidence other than lung, liver and bone in Mayak workers: 1948-2004. Br. J. Cancer.

[15] Azimzadeh O., Sievert W., Sarioglu H., Yentrapalli R., Barjaktarovic Z., Sriharshan A., Ueffing M., Janik D., Aichler M., Atkinson M.J. (2013). PPAR Alpha: A Novel Radiation Target in Locally Exposed Mus musculus Heart Revealed by Quantitative Proteomics. J. Proteome Res..

[16] Barjaktarovic Z., Shyla A., Azimzadeh O., Schulz S., Haagen J., Dorr W., Sarioglu H., Atkinson M.J., Zischka H., Tapio S. (2013). Ionising radiation induces persistent alterations in the cardiac mitochondrial function of C57BL/6 mice 40 weeks after local heart exposure. Radiother. Oncol..

[17] Barjaktarovic Z., Schmaltz D., Shyla A., Azimzadeh O., Schulz S., Haagen J., Dorr W., Sarioglu H., Schafer A., Atkinson M.J. (2011). Radiation-induced signaling results in mitochondrial impairment in mouse heart at 4 weeks after exposure to X-rays. PLoS ONE.

[18] Azimzadeh O., Azizova T., Merl-Pham J., Subramanian V., Bakshi M.V., Moseeva M., Zubkova O., Hauck S.M., Anastasov N., Atkinson M.J. (2017). A dose-dependent perturbation in cardiac energy metabolism is linked to radiation-induced ischemic heart disease in Mayak nuclear workers. Oncotarget.

[19] Hebert A.S., Dittenhafer-Reed K.E., Yu W., Bailey D.J., Selen E.S., Boersma M.D., Carson J.J., Tonelli M., Balloon A.J., Higbee A.J. (2013). Calorie restriction and SIRT3 trigger global reprogramming of the mitochondrial protein acetylome. Mol. Cell.

[20] Hirschey M.D., Shimazu T., Goetzman E., Jing E., Schwer B., Lombard D.B., Grueter C.A., Harris C., Biddinger S., Ilkayeva O.R. (2010). SIRT3 regulates mitochondrial fatty-acid oxidation by reversible enzyme deacetylation. Nature.

[21] Hirschey M.D., Shimazu T., Jing E., Grueter C.A., Collins A.M., Aouizerat B., Stancakova A., Goetzman E., Lam M.M., Schwer B. (2011). SIRT3 deficiency and mitochondrial protein hyperacetylation accelerate the development of the metabolic syndrome. Mol. Cell.

[22] Parodi-Rullan R.M., Chapa-Dubocq X.R., Javadov S. (2018). Acetylation of Mitochondrial Proteins in the Heart: The Role of SIRT3. Front. Physiol..

[23] Fukushima A., Lopaschuk G.D. (2016). Acetylation control of cardiac fatty acid beta-oxidation and energy metabolism in obesity, diabetes, and heart failure. Biochim. Biophys. Acta.

[24] Haigis M.C., Mostoslavsky R., Haigis K.M., Fahie K., Christodoulou D.C., Murphy A.J., Valenzuela D.M., Yancopoulos G.D., Karow M., Blander G. (2006). SIRT4 inhibits glutamate dehydrogenase and opposes the effects of calorie restriction in pancreatic beta cells. Cell.

[25] Anderson K.A., Huynh F.K., Fisher-Wellman K., Stuart J.D., Peterson B.S., Douros J.D., Wagner G.R., Thompson J.W., Madsen A.S., Green M.F. (2017). SIRT4 Is a Lysine Deacylase that Controls Leucine Metabolism and Insulin Secretion. Cell Metab..

[26] Frye R.A. (2000). Phylogenetic classification of prokaryotic and eukaryotic Sir2-like proteins. Biochem. Biophys. Res. Commun..

[27] Saunders L.R., Verdin E. (2007). Sirtuins: Critical regulators at the crossroads between cancer and aging. Oncogene.

[28] Nemoto S., Fergusson M.M., Finkel T. (2005). SIRT1 functionally interacts with the metabolic regulator and transcriptional coactivator PGC-1{alpha}. J. Biol. Chem..

[29] Vega R.B., Huss J.M., Kelly D.P. (2000). The coactivator PGC-1 cooperates with peroxisome proliferator-activated receptor alpha in transcriptional control of nuclear genes encoding mitochondrial fatty acid oxidation enzymes. Mol. Cell. Biol..

[30] Canto C., Gerhart-Hines Z., Feige J.N., Lagouge M., Noriega L., Milne J.C., Elliott P.J., Puigserver P., Auwerx J. (2009). AMPK regulates energy expenditure by modulating NAD+ metabolism and SIRT1 activity. Nature.

[31] Kong X., Wang R., Xue Y., Liu X., Zhang H., Chen Y., Fang F., Chang Y. (2010). Sirtuin 3, a new target of PGC-1alpha, plays an important role in the suppression of ROS and mitochondrial biogenesis. PLoS ONE.

[32] Zhang X., Ren X., Zhang Q., Li Z., Ma S., Bao J., Li Z., Bai X., Zheng L., Zhang Z. (2016). PGC-1alpha/ERRalpha-Sirt3 Pathway Regulates DAergic Neuronal Death by Directly Deacetylating SOD2 and ATP Synthase beta. Antioxid. Redox Signal..

[33] Shi L., Tu B.P. (2015). Acetyl-CoA and the regulation of metabolism: Mechanisms and consequences. Curr. Opin. Cell Biol..

[34] Lo Sasso G., Schlage W.K., Boue S., Veljkovic E., Peitsch M.C., Hoeng J. (2016). The Apoe(-/-) mouse model: A suitable model to study cardiovascular and respiratory diseases in the context of cigarette smoke exposure and harm reduction. J. Transl. Med..

[35] Mancuso M., Pasquali E., Braga-Tanaka I., Tanaka S., Pannicelli A., Giardullo P., Pazzaglia S., Tapio S., Atkinson M.J., Saran A. (2015). Acceleration of atherogenesis in ApoE ^-/-^ mice exposed to acute or low-dose-rate ionizing radiation. Oncotarget.

[36] Kumarathasan P., Vincent R., Blais E., Saravanamuthu A., Gupta P., Wyatt H., Mitchel R., Hannan M., Trivedi A., Whitman S. (2013). Cardiovascular changes in atherosclerotic ApoE-deficient mice exposed to Co60 (gamma) radiation. PLoS ONE.

[37] Calvo S.E., Clauser K.R., Mootha V.K. (2016). MitoCarta2.0: An updated inventory of mammalian mitochondrial proteins. Nucleic Acids Res..

[38] Chan S.H., Wu K.L., Chang A.Y., Tai M.H., Chan J.Y. (2009). Oxidative impairment of mitochondrial electron transport chain complexes in rostral ventrolateral medulla contributes to neurogenic hypertension. Hypertension.

[39] Karamanlidis G., Lee C.F., Garcia-Menendez L., Kolwicz S.C., Suthammarak W., Gong G., Sedensky M.M., Morgan P.G., Wang W., Tian R. (2013). Mitochondrial complex I deficiency increases protein acetylation and accelerates heart failure. Cell Metab..

[40] Jeninga E.H., Schoonjans K., Auwerx J. (2010). Reversible acetylation of PGC-1: Connecting energy sensors and effectors to guarantee metabolic flexibility. Oncogene.

[41] Barger P.M., Brandt J.M., Leone T.C., Weinheimer C.J., Kelly D.P. (2000). Deactivation of peroxisome proliferator-activated receptor-alpha during cardiac hypertrophic growth. J. Clin. Investig..

[42] Papiez A., Azimzadeh O., Azizova T., Moseeva M., Anastasov N., Smida J., Tapio S., Polanska J. (2018). Integrative multiomics study for validation of mechanisms in radiation-induced ischemic heart disease in Mayak workers. PLoS ONE.

[43] Polevoda B., Sherman F. (2002). The diversity of acetylated proteins. Genome Biol..

[44] Matsushima S., Sadoshima J. (2015). The role of sirtuins in cardiac disease. Am. J. Physiol. Heart Circ. Physiol..

[45] Ali I., Conrad R.J., Verdin E., Ott M. (2018). Lysine Acetylation Goes Global: From Epigenetics to Metabolism and Therapeutics. Chem. Rev..

[46] Zhao S., Xu W., Jiang W., Yu W., Lin Y., Zhang T., Yao J., Zhou L., Zeng Y., Li H. (2010). Regulation of cellular metabolism by protein lysine acetylation. Science.

[47] Wang Q., Zhang Y., Yang C., Xiong H., Lin Y., Yao J., Li H., Xie L., Zhao W., Yao Y. (2010). Acetylation of metabolic enzymes coordinates carbon source utilization and metabolic flux. Science.

[48] Chen T., Liu J., Li N., Wang S., Liu H., Li J., Zhang Y., Bu P. (2015). Mouse SIRT3 attenuates hypertrophy-related lipid accumulation in the heart through the deacetylation of LCAD. PLoS ONE.

[49] Grillon J.M., Johnson K.R., Kotlo K., Danziger R.S. (2012). Non-histone lysine acetylated proteins in heart failure. Biochim. Biophys. Acta.

[50] Lewin M.B., Timiras P.S. (1984). Lipid changes with aging in cardiac mitochondrial membranes. Mech. Ageing Dev..

[51] Hafner A.V., Dai J., Gomes A.P., Xiao C.Y., Palmeira C.M., Rosenzweig A., Sinclair D.A. (2010). Regulation of the mPTP by SIRT3-mediated deacetylation of CypD at lysine 166 suppresses age-related cardiac hypertrophy. Aging.

[52] Sundaresan N.R., Gupta M., Kim G., Rajamohan S.B., Isbatan A., Gupta M.P. (2009). Sirt3 blocks the cardiac hypertrophic response by augmenting Foxo3a-dependent antioxidant defense mechanisms in mice. J. Clin. Investig..

[53] Cencioni C., Spallotta F., Mai A., Martelli F., Farsetti A., Zeiher A.M., Gaetano C. (2015). Sirtuin function in aging heart and vessels. J. Mol. Cell. Cardiol..

[54] Donato A.J., Magerko K.A., Lawson B.R., Durrant J.R., Lesniewski L.A., Seals D.R. (2011). SIRT-1 and vascular endothelial dysfunction with ageing in mice and humans. J. Physiol..

[55] Ghosh S., Zhou Z. (2015). SIRTain regulators of premature senescence and accelerated aging. Protein Cell.

[56] Grabowska W., Sikora E., Bielak-Zmijewska A. (2017). Sirtuins, a promising target in slowing down the ageing process. Biogerontology.

[57] Shimizu I., Minamino T. (2019). Cellular senescence in cardiac diseases. J. Cardiol..

[58] Li Y., Ma Y., Song L., Yu L., Zhang L., Zhang Y., Xing Y., Yin Y., Ma H. (2018). SIRT3 deficiency exacerbates p53/Parkinmediated mitophagy inhibition and promotes mitochondrial dysfunction: Implication for aged hearts. Int. J. Mol. Med..

[59] Lin S., Wang Y., Zhang X., Kong Q., Li C., Li Y., Ding Z., Liu L. (2016). HSP27 Alleviates Cardiac Aging in Mice via a Mechanism Involving Antioxidation and Mitophagy Activation. Oxid. Med. Cell. Longev..

[60] Lu J., Zhang H., Chen X., Zou Y., Li J., Wang L., Wu M., Zang J., Yu Y., Zhuang W. (2017). A small molecule activator of SIRT3 promotes deacetylation and activation of manganese superoxide dismutase. Free Radic. Biol. Med..

[61] Tao R., Coleman M.C., Pennington J.D., Ozden O., Park S.H., Jiang H., Kim H.S., Flynn C.R., Hill S., Hayes McDonald W. (2010). Sirt3-mediated deacetylation of evolutionarily conserved lysine 122 regulates MnSOD activity in response to stress. Mol. Cell.

[62] Anderson E.J., Katunga L.A., Willis M.S. (2012). Mitochondria as a source and target of lipid peroxidation products in healthy and diseased heart. Clin. Exp. Pharmacol. Physiol..

[63] Chen T., Dai S.H., Li X., Luo P., Zhu J., Wang Y.H., Fei Z., Jiang X.F. (2018). Sirt1-Sirt3 axis regulates human blood-brain barrier permeability in response to ischemia. Redox. Biol..

[64] Kwon S., Seok S., Yau P., Li X., Kemper B., Kemper J.K. (2017). Obesity and aging diminish sirtuin 1 (SIRT1)-mediated deacetylation of SIRT3, leading to hyperacetylation and decreased activity and stability of SIRT3. J. Biol. Chem..

[65] Carnevale I., Pellegrini L., D’Aquila P., Saladini S., Lococo E., Polletta L., Vernucci E., Foglio E., Coppola S., Sansone L. (2017). SIRT1-SIRT3 Axis Regulates Cellular Response to Oxidative Stress and Etoposide. J. Cell. Physiol..

[66] Verdin E., Hirschey M.D., Finley L.W., Haigis M.C. (2010). Sirtuin regulation of mitochondria: Energy production, apoptosis, and signaling. Trends Biochem. Sci..

[67] Khan S.A., Sathyanarayan A., Mashek M.T., Ong K.T., Wollaston-Hayden E.E., Mashek D.G. (2015). ATGL-catalyzed lipolysis regulates SIRT1 to control PGC-1alpha/PPAR-alpha signaling. Diabetes.

[68] Barjaktarovic Z., Kempf S.J., Sriharshan A., Merl-Pham J., Atkinson M.J., Tapio S. (2015). Ionizing radiation induces immediate protein acetylation changes in human cardiac microvascular endothelial cells. J. Radiat. Res..

[69] Tyanova S., Temu T., Sinitcyn P., Carlson A., Hein M.Y., Geiger T., Mann M., Cox J. (2016). The Perseus computational platform for comprehensive analysis of (prote)omics data. Nat. Methods.

[70] Spellman P.T., Sherlock G., Zhang M.Q., Iyer V.R., Anders K., Eisen M.B., Brown P.O., Botstein D., Futcher B. (1998). Comprehensive identification of cell cycle-regulated genes of the yeast Saccharomyces cerevisiae by microarray hybridization. Mol. Biol. Cell.

[71] Szklarczyk D., Gable A.L., Lyon D., Junge A., Wyder S., Huerta-Cepas J., Simonovic M., Doncheva N.T., Morris J.H., Bork P. (2019). STRING v11: Protein-protein association networks with increased coverage, supporting functional discovery in genome-wide experimental datasets. Nucleic Acids. Res..

[72] Kramer A., Green J., Pollard J., Tugendreich S. (2014). Causal analysis approaches in Ingenuity Pathway Analysis. Bioinformatics.

