# Supplementary material for: Hyperacetylation of Cardiac Mitochondrial Proteins Is Associated with Metabolic Impairment and Sirtuin Downregulation after Chronic Total Body Irradiation of ApoE -/- Mice
Source: Int J Mol Sci. 2019 Oct 22;20(20):5239. doi: 10.3390/ijms20205239 (PMC6829468; doi:10.3390/ijms20205239)
Supplement: Supplementary file 1 [file ijms-20-05239-s001.zip › ijms-624297-for publish-Supplementary Figures.docx]

Supplementary Figures

Hyperacetylation of Cardiac Mitochondrial Proteins is Associated with Metabolic Impairment and Sirtuin Downregulation after Chronic Total Body Irradiation of ApoE ^-/-^ Mice

Zarko Barjaktarovic ^1, 2^, Juliane Merl-Pham ^3^, Ignacia Braga-Tanaka ^4^, Satoshi Tanaka ^4^, Stefanie M. Hauck ^3^, Anna Saran ^5^, Mariateresa Mancuso ^5^, Michael J. Atkinson ^1, 6^, Soile Tapio ^1^ and Omid Azimzadeh ^1,^ *


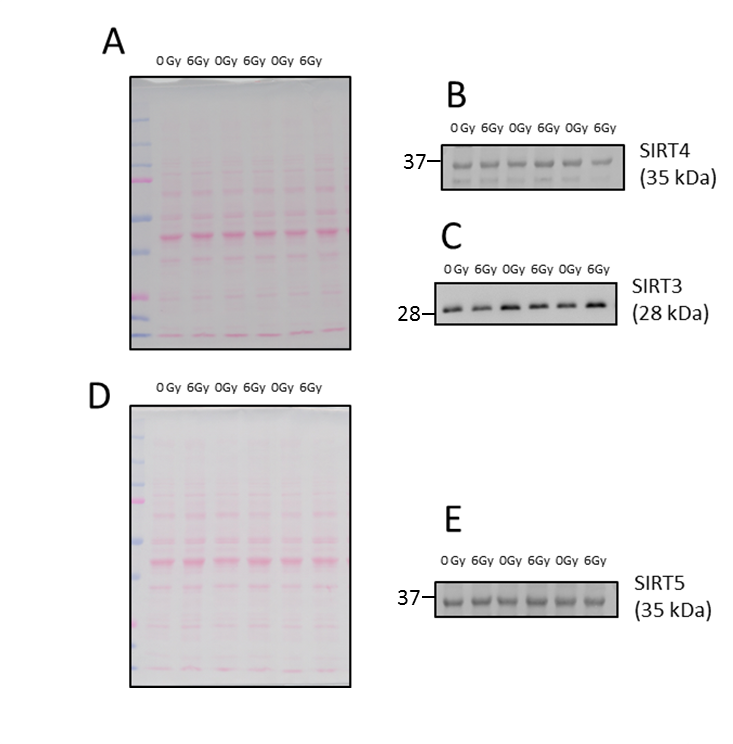


**Figure 1.** Full-length images of Ponceau S staining and antibody detections of replicates for SIRT3, SIRT4 and SIRT5.


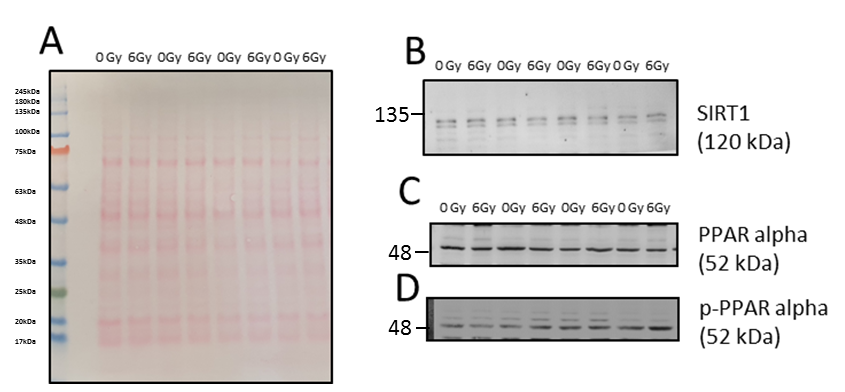


**Figure 2.** Full-length images of Ponceau S staining and antibody detections of replicates for SIRT1, PPAR alpha and p-PPAR alpha.


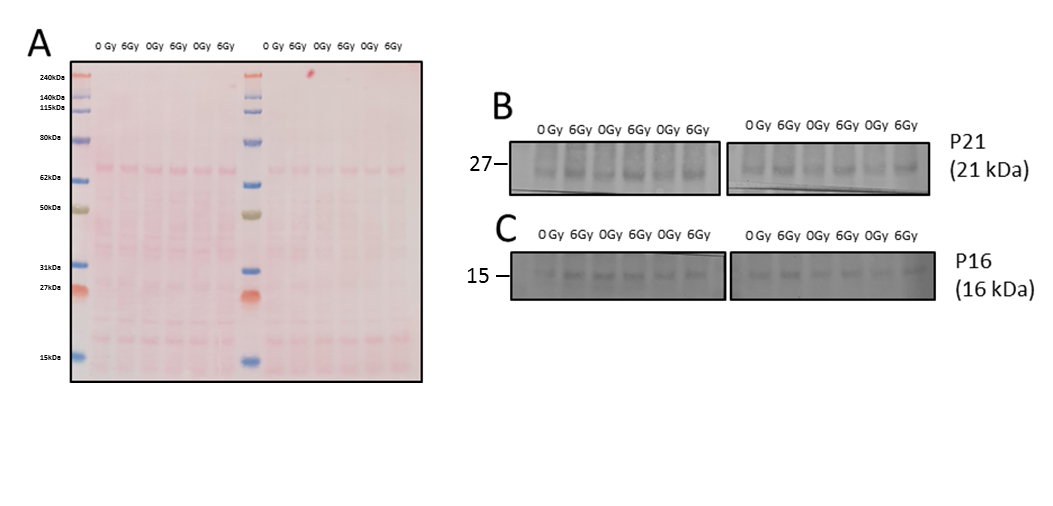


**Figure 3.** Full-length images of Ponceau S staining and antibody detections of replicates for p21and p16.


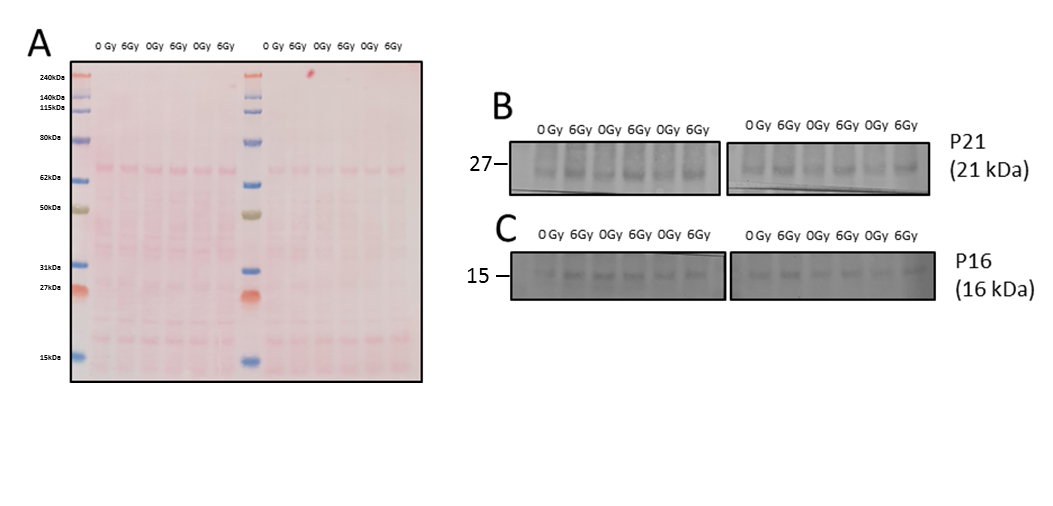


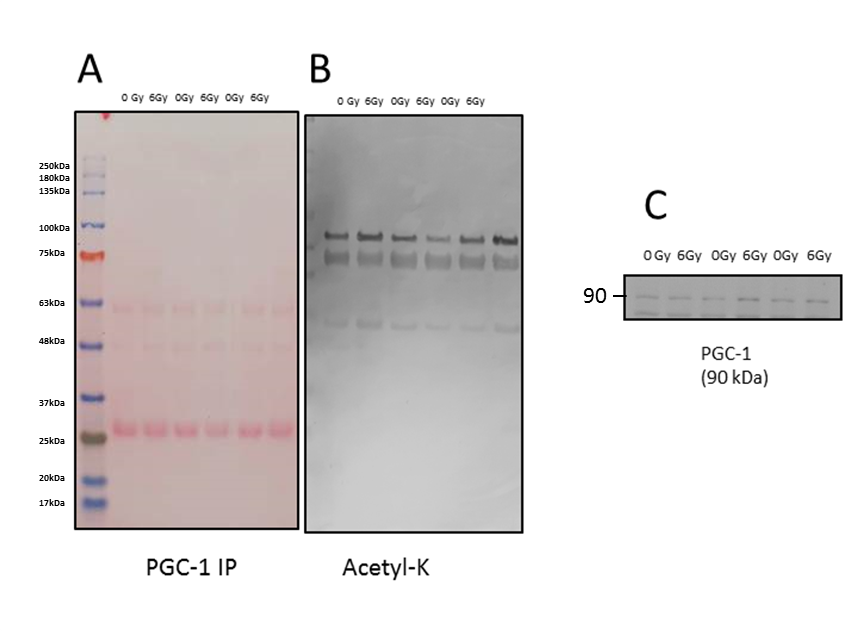


**Figure 4.** Full-length images of Ponceau S staining and antibody detections of replicates for PGC-1 and Acetylated lysine (Acetyl-K).


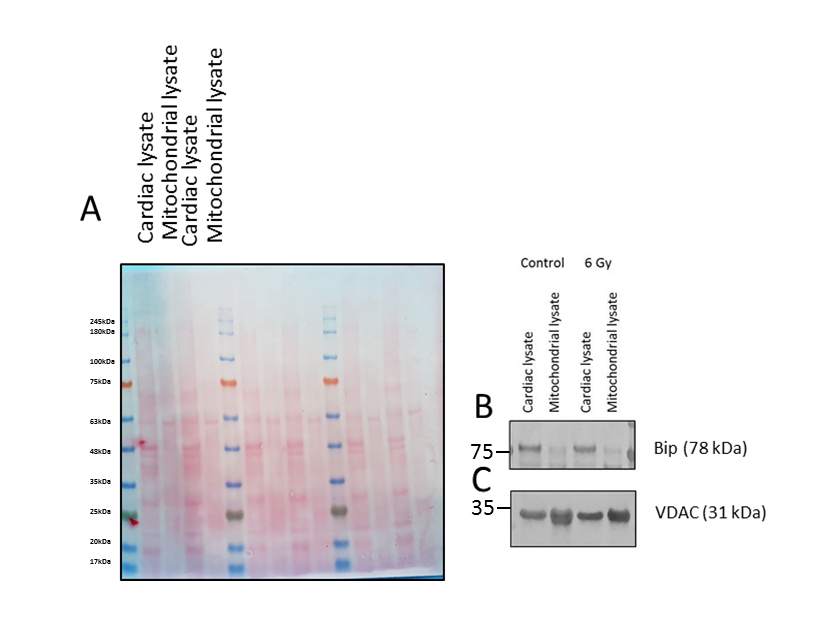


**Figure 5.** Full-length images of Ponceau S staining and antibody detections of replicates for markers for ER (BiP) and mitochondria (VDAC).

| 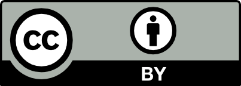 | © 2019 by the authors. Submitted for possible open access publication under the terms and conditions of the Creative Commons Attribution (CC BY) license (http://creativecommons.org/licenses/by/4.0/). |
| --- | --- |
